# Long-Term Durability and Public Health Impact of City-Wide *w*Mel *Wolbachia* Mosquito Releases in Niterói, Brazil, During a Dengue Epidemic Surge

**DOI:** 10.3390/tropicalmed10090237

**Published:** 2025-08-25

**Authors:** Katherine L. Anders, Gabriel Sylvestre Ribeiro, Renato da Silva Lopes, Pilar Amadeu, Thiago Rodrigues da Costa, Thais Irene Souza Riback, Karlos Diogo de Melo Chalegre, Wesley Pimentel de Oliveira, Cátia Cabral da Silva, Marcos Vinicius Ferreira Mendes Blanco, Ana Lucia Fontes Eppinghaus, Fabio Villas Boas, Tibor Frossard, Benjamin R. Green, Scott L. O’Neill, Peter A. Ryan, Cameron P. Simmons, Luciano A. Moreira

**Affiliations:** 1 World Mosquito Program, Melbourne, VIC 3800, Australiaben.green@worldmosquito.org (B.R.G.); scott.oneill@worldmosquito.org (S.L.O.); peter.ryan@worldmosquito.org (P.A.R.); cameron.simmons@worldmosquito.org (C.P.S.); 2 School of Public Health and Preventive Medicine, Monash University, Melbourne, VIC 3004, Australia; 3 World Mosquito Program, Rio de Janeiro 21040-900, Rio de Janeiro, Brazil; gabriel.sylvestre@wolbito.com (G.S.R.); renato.lopes@wmprojects.org (R.d.S.L.); pilar.amadeu@wmprojects.org (P.A.); thiago.costa@wolbito.com (T.R.d.C.); wesley.oliveira@wmprojects.org (W.P.d.O.); catia.cabral@wmprojects.org (C.C.d.S.); luciano.andrade@fiocruz.br (L.A.M.); 4 City Health Secretariat, Niterói 24020-206, Rio de Janeiro, Brazil; 5 Fiocruz–Instituto René Rachou, Belo Horizonte 30190-002, Minas Gerais, Brazil

**Keywords:** dengue, *Wolbachia*, *Aedes aegypti*, vector control, innovative tools, biocontrol, mosquito releases, outbreak prevention

## Abstract

In 2024, the Americas experienced the largest dengue outbreak on record and Brazil was among the worst affected countries, reporting 6.6 million cases and 6200 deaths. We report the long-term entomological and epidemiological effectiveness of city-wide deployment of *w*Mel-strain *Wolbachia*-infected *Aedes aegypti* in Niterói, a city of half a million people in Rio de Janeiro state, where *Wolbachia* releases across three-quarters of the urban population in 2017–2019 were expanded to remaining populated areas in 2023. *w*Mel was durably established at ≥95% prevalence in *Ae. aegypti* populations throughout Niterói four years post-release, and up to seven years in the earliest release sites. Notified dengue case incidence in Niterói was 89% lower following *Wolbachia* releases, compared to the 10-year pre-intervention period of 2007–2016. Dengue incidence in Niterói in 2024, during a period of record high incidence in Brazil and the region, was 374 per 100,000 population, substantially lower than overall in Rio de Janeiro state (1884 per 100,000) and nationwide in Brazil (3157 per 100,000). Our findings show that city-wide *Wolbachia* coverage in Niterói provided sustained population-level reduction in dengue incidence throughout the five years post-intervention, including during the 2024 epidemic surge, averting an estimated three-quarters of the dengue case burden that may otherwise have been expected in Niterói in 2024.

## 1. Introduction

Dengue has been a public health problem in the tropical world for decades. A global dengue surge in 2024 saw in excess of 14 million cases and 10 thousand deaths reported worldwide [1]—more than doubling the largest epidemics previously recorded in 2023 and 2019. The Americas region accounted for the vast majority of reported dengue cases and deaths in 2024 [1]. Brazil was among the worst affected countries, reporting 6.6 million cases and 6200 deaths [2], and the projected economic impact on the country was estimated at US$3 billion well before the true magnitude of the epidemic was evident [3].

Arboviral disease prevention and control efforts in the Americas and elsewhere have long emphasized an intersectoral approach integrating epidemiological and entomological surveillance, chemical and environmental control of mosquito populations, together with effective clinical management [4]. The success of these efforts in mitigating dengue epidemic spread is hampered by insecticide resistance, the limited feasibility of scaling and sustaining vector source reduction activities in complex urban environments, and limited funding for these recurrent activities within public health budgets. Vaccines for dengue and chikungunya have been authorized for use in several countries since 2022, and in February 2024 Brazil became the first country to launch a public dengue vaccination program, targeting 10 to 14 year-olds in the municipalities with highest case incidence [5]. Brazil has also been at the forefront of evidence generation for *Wolbachia*-based arboviral disease control, a self-sustaining strategy that involves time-limited releases of *Aedes aegypti* mosquitoes carrying the endosymbiotic insect bacterium *Wolbachia*, which spreads into the local *Ae. aegypti* population and significantly reduces the mosquitoes’ capacity to transmit dengue and other viruses [6,7,8,9,10].

**Figure 1:**
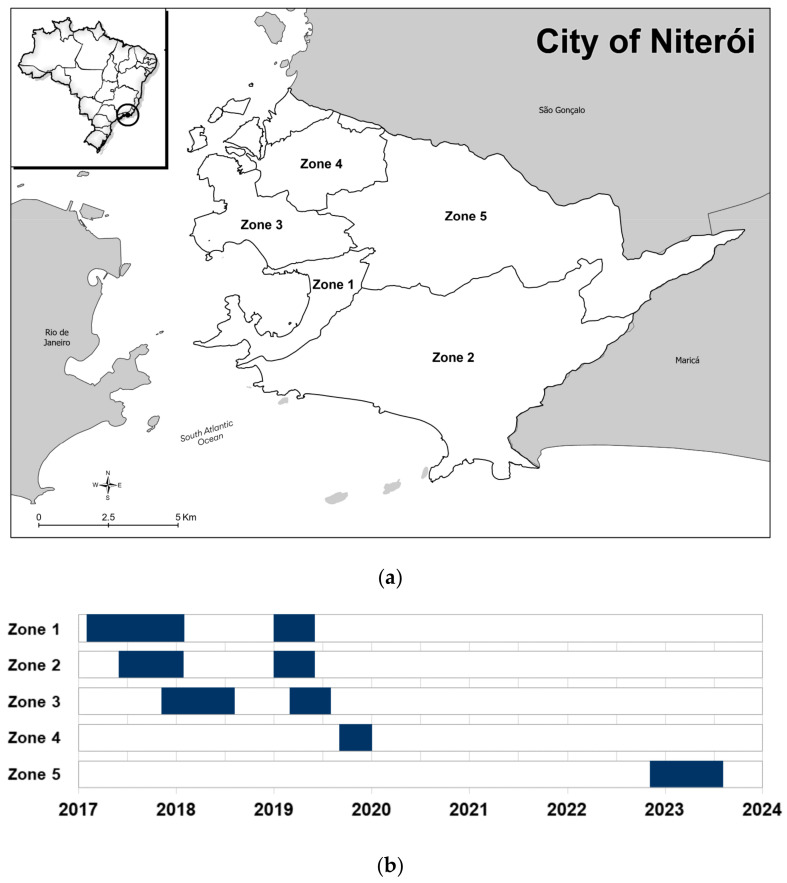
*Wolbachia* release areas and timeline in Niterói, Brazil. (**a**) Map of Niterói showing the release zones, and the location of Niterói within Rio de Janeiro State and Brazil (inset). (**b**) Timeline of *w*Mel-infected *Ae. aegypti* releases in Zone 1 (February 2017–January 2018 and January–May 2019); Zone 2 (June 2017–January 2018 and January–May 2019); Zone 3 (November 2017–July 2018 and March–July 2019); Zone 4 (September–December 2019); and Zone 5 (November 2022–July 2023).

Following successful pilot releases of *w*Mel-strain *Wolbachia*-infected mosquitoes in the adjacent municipalities of Rio de Janeiro [11] and Niterói [12,13] in 2014 and 2015, respectively, phased expanded releases were conducted in the two cities between 2017 and 2019. Early monitoring showed evidence of significant reductions in dengue and chikungunya incidence in *Wolbachia*-treated areas in both cities, despite incomplete and spatially variable levels of introgression within the first 1–2 years post-release [13,14]—consistent with an accumulating body of evidence from randomized and non-randomized field trials in Australia, Asia and Latin America [15,16,17,18,19,20]. Economic modeling studies in Brazil [21] and elsewhere [22,23] concur on the long-term cost-effectiveness of *Wolbachia-*based disease control under scenarios of scaled programmatic deployment in high-burden, populous cities, however challenges remain with financing the upfront costs of initiating and scaling *Wolbachia* release programs. A better understanding of the durability and long-term public health impact of *Wolbachia* deployments in different settings can help inform scale-up plans, including optimal integration with vaccination programs and integrated vector management efforts. The long-term durability of *w*Mel-*Wolbachia* in the field has been demonstrated a decade post-release in northern Queensland, Australia [24,25], and up to four and six years after the earliest releases in Colombia [26] and Indonesia [19], respectively, but more variable outcomes have been reported in other settings [27,28]. Here we report the long-term entomological and epidemiological outcomes of city-wide *Wolbachia* deployments in Niterói (Figure 1), a Brazilian city of half a million people, which demonstrate sustained public health benefits in the context of an unprecedented dengue outbreak in Brazil.

## 2. Materials and Methods

### 2.1. Ethics Statement

Approval to release *Wolbachia*-infected *Ae. aegypti* mosquitoes into urban areas was obtained from three Brazilian governmental bodies: the National Agency of Sanitary Surveillance (ANVISA); the Ministry of Agriculture, Livestock and Supply (MAPA); and the Brazilian Institute of Environment and Renewable Natural Resources (IBAMA), which issued a Temporary Special Registry (Registro Especial Temporário (RET), nr. 0551716178/2017). Ethical approval was also obtained from the National Commission for Research Ethics (CONEP—CAAE 59175616.2.0000.0008).

### 2.2. Mosquito Production

The Rio *w*Mel-infected *Ae. aegypti* line (*w*MelRio) described in [11] was used for both the initial and expanded releases in Niterói. Briefly, this *w*MelRio line was created originally by backcrossing *w*Mel-infected *Ae. aegypti* imported from Australia (IBAMA license 11BR005873/DF) with Rio de Janeiro *Ae. aegypti* to produce a *Wolbachia*-infected line with a Rio de Janeiro genetic background [11]. The *w*MelRio line was outcrossed to wild-type material collected from ovitraps in untreated areas of Niterói every six months up to the end of 2023 when the colony was closed. Wild-type males reared from the ovitrap collections were added to the colony in the proportion of 25% wild-type males to 75% *w*Mel-infected males (cages with 3000 individuals) and their eggs were collected through four gonotrophic cycles. *w*Mel infection frequency in the release-production colony was monitored weekly and kdr genotyping was performed monthly to monitor pyrethroid insecticide resistance in the release line, as described previously [13]. Immature stages for adult releases were reared at a density of approximately 2.75 larvae/mL and fed a diet of fish food: liver powder: yeast extract (4:3:1) as described previously [13]. For Zone 5 releases, a larval/pupal separator (an adapted format of the glass pupae separator www.johnwhock.com/products/laboratory-equipment/larval-pupal-separator (accessed on 1 August 2025) John W. Hock Company, Gainesville, FL, USA) was used to sort and collect the pupae, which were then transferred to release devices (220 pupae per device). These release devices were hexagon tubes approximately 60 mm in diameter and 170 mm in length, with a removable well on top. Adults were allowed to emerge for 5–6 days and were maintained on a 10% sugar solution for 12–36 h prior to releases. The release tubes were then put into boxes and organized in vehicles for field releases. Five release tubes from each release vehicle were retained for quality control monitoring. Tubes were maintained throughout the release run and then returned to the laboratory and the surviving adult mosquitoes were sexed and counted.

### 2.3. wMel Deployment in Niterói

Releases of *w*Mel-*Ae. aegypti* throughout three-quarters of the urban population of Niterói (release zones 1–4) in 2017–2019 have been reported previously (Figure 1) [13]. For the expanded releases in the remaining urban areas of Niterói (zone 5), the density of release points was adjusted for the residential population in each neighborhood, with the aim of reaching a cumulative total of 200 mosquitoes per person. The release tubes containing an average of 200 mosquitoes, males and females, were distributed in routes and released from a vehicle, or on foot in areas where the vehicle could not enter due to local restrictions. For these on-foot releases, a partnership with local vector control agents from the municipality was created and allowed the coverage of the whole territory. Weekly releases were conducted from November 2022 to July 2023, with between 28 and 32 release weeks per neighborhood.

### 2.4. wMel Monitoring

Long-term monitoring of *w*Mel prevalence in local *Ae. aegypti* populations in zones 1–4 used adult mosquitoes collected with BG Sentinel traps until 2020 as described previously [13], and then with aspirator collections from 2021 onwards (Improved Prokopack Aspirator Model 1419, John W. Hock Company, Gainesville, FL, USA). In Zone 5, monitoring used mosquito eggs instead of adults, for the purpose of integrating field collections with the routine vector monitoring activities conducted by the Niterói municipal health authorities. *Ae. aegypti* eggs were sampled using an existing network of ovitraps (approximately one trap per 500 × 500 m grid) containing a wood paddle where the females lay the eggs. Fortnightly, these traps were collected, the paddles were left to dry for 48 h and then put into cups with water and fish food for hatching. After 4–5 days, L4 larvae were identified for species and maximum 20 larvae per trap were randomly selected for diagnostics, stored in 80% ethanol and then individually quantitative polymerase chain reaction (qPCR) processed for *w*Mel-strain *Wolbachia* detection [13].

### 2.5. Dengue Case Notifications and Population Data

Dengue cases are notified to the Brazilian national disease surveillance system (SINAN) on the basis of a clinical case definition [29]; only a minority of cases are laboratory confirmed. Line listed deidentified data was obtained from the SINAN system through the Health Secretariat of Niterói, on notified dengue cases resident in Niterói for the period January 2007 to September 2024 and on notified chikungunya and Zika cases for the period January 2015 to September 2024. A ten-year pre-intervention period was used for the dengue data to ensure comparability over time, because a new version of SINAN software (Version 5.0.0.0/Patch 5.3.0.0) was implemented nationwide in January 2007. No chikungunya or Zika notification data is available prior to 2015. Data was aggregated to monthly dengue case counts by *Wolbachia* release zone (1–5), based on cases’ neighborhood of residence.

National data on annual notified dengue case numbers by municipality of residence for all of Brazil was also downloaded from the publicly accessible SINAN database (https://datasus.saude.gov.br/informacoes-de-saude-tabnet/) on 11 February 2025, for the years 2007–2024.

Data on the residential population of municipalities in Brazil was obtained from the 2000, 2010 and 2022 Brazilian censuses (IBGE: https://sidra.ibge.gov.br/pesquisa/censo-demografico, accessed on 11 February 2025). Census data from 2000 and 2010 was available by neighborhood of residence for Niterói and was aggregated to estimate the population of each release zone, using 2000 data for the years 2007–2009 and 2010 data for the years 2010–2019. We estimated the release zone populations in 2020–2024 by applying the 2010 zone-level population distribution to the Niterói 2022 census population.

### 2.6. Statistical Methods

Per capita annual dengue incidence in Niterói was calculated as the annual number of notified cases per 1000 inhabitants. The *Wolbachia* intervention effect was estimated by interrupted time series (ITS) analysis [30], implemented as reported previously [19,20] using mixed-effect negative binomial regression to model the monthly count of dengue case notifications in each release zone as a function of *Wolbachia* release status (before, during or after releases), with an offset for population size, calendar month as a fixed-effect covariate, and release zone modeled as a random effect. The model output is the dengue incidence rate ratio in the post- or during-release periods compared with pre-release periods, adjusted for seasonality. Robust standard errors were used by specifying the vce (cluster *release_zone*) option in StataSE version 18.0 (StataCorp LLC, College Station, TX, USA) to account for non-independence of observations within release zones.

## 3. Results

### 3.1. City-Wide Coverage and Long-Term Stability of Wolbachia in the Niterói Ae. aegypti Mosquito Population

**Figure 2:**
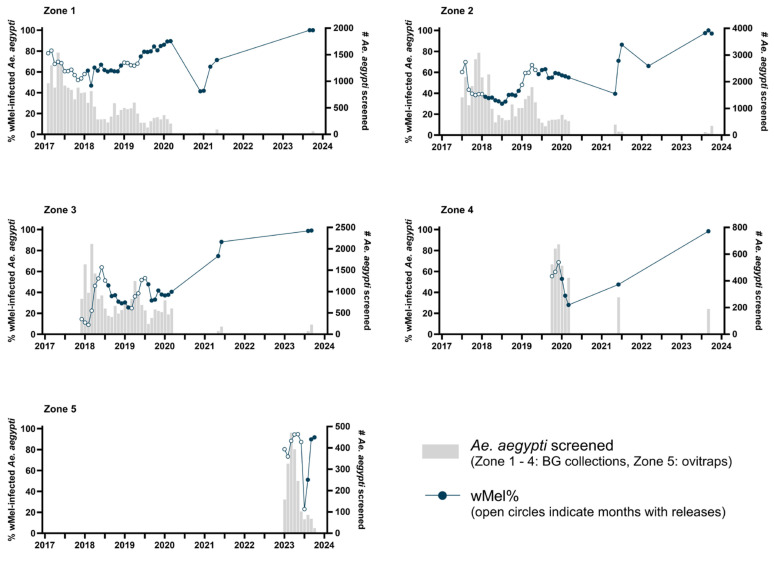
*w*Mel infection prevalence in *Aedes aegypti* mosquitoes collected from each release zone, during and after *Wolbachia* releases. Circle markers represent the aggregate *w*Mel infection prevalence in each zone in each calendar month from January 2017 to October 2023. Open circles indicate months when *Wolbachia* releases took place in any part of that zone; filled circles are months with no releases. Bars show the number of *Ae. aegypti* tested for *w*Mel infection by qPCR. In zones 1–4 adult *Ae. aegypti* were collected with BG traps, and in zone 5 ovitraps were used to collect eggs that were reared to larvae for species identification and *w*Mel testing. Monitoring results with <20 *Ae. aegypti* tested were excluded from the graphs (Zone 1: *n* = 4 observations; Zone 2: *n* = 6; Zone 3: *n* = 4; Zone 4: *n* = 0; Zone 5: *n* = 3).

Following the phased deployment of *w*Mel-infected *Ae. aegypti* throughout three-quarters of Niterói’s urban population between February 2017 and December 2019 (release zones 1–4; population 371,000) [13], releases of the same *w*Mel-infected *Ae. aegypti* line were expanded to the remaining urban areas of the city (zone 5; population 111,000) between November 2022 and July 2023 (Figure 1; Table S1). In zone 5, an estimated 21.3 million mosquitoes were released as adults from moving vehicles over 32 release weeks and the establishment of *w*Mel in the local *Ae. aegypti* population was monitored in larvae reared from ovitrap collections undertaken by municipal health authorities as part of routine vector surveillance activities. *w*Mel prevalence in zone 5 was 51% (44 *w*Mel-positive of 86 larvae tested from 15 traps) by August 2023, 3 months post-release and 92% (22/24 larvae from 10 traps) at last monitoring in October 2023, 5 months post-release (Figure 2).

Long-term monitoring of the invasion and stability of *w*Mel in the *Ae. aegypti* populations in the 2017–2019 release areas (zones 1–4) was conducted between 2021 and 2023, using BG Sentinel traps and mechanical aspirators to catch and screen adult mosquitoes from across the release areas, consistent with the initial monitoring up to March 2020 reported previously from these areas [13]. At last monitoring in July–October 2023, four years after the end of releases, between 97% and 100% of the *Ae. aegypti* screened in each zone were *w*Mel-positive (Figure 2. *n* = 1096 total *Ae. aegypti* screened; 80–527 per zone).

**Figure 3:**
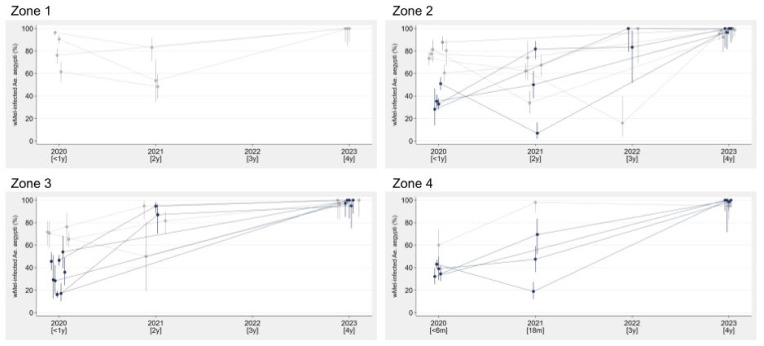
*Wolbachia* invasion profiles in individual neighborhoods during long-term monitoring up to four years post-release. *w*Mel-infected *Ae. aegypti* mosquito releases were completed in May (Zones 1 & 2), July (Zone 3) or December (Zone 4) 2019. Markers represent the percentage of adult *Ae. aegypti* collected by BG trap or aspiration that were positive for *w*Mel *Wolbachia* by PCR; vertical lines show the 95% confidence interval for the sample proportion, calculated by the Clopper-Pearson exact method. Collections were conducted across 1–3 months each year in each neighborhood, and aggregated by year to account for different collection months and relatively small sample sizes. Observations where <10 *Ae. aegypti* were screened from a neighborhood in a year were excluded. Monitoring in 2020 was between January and March, and these data have been reported previously [13]. Of 32 neighborhoods in zones 1–4, all but two had *w*Mel% results in quarter 1 2020; 26 of these neighborhoods were monitored in 2023 (*n* > 10 *Ae. aegypti*), and 18 have additional monitoring results from 2021 and/or 2022. Gray lines indicate neighborhoods where *w*Mel was already well-established (≥60%) in quarter 1 2020, within 6–12 months of releases, and black lines are neighborhoods where *w*Mel prevalence was <60% in short-term monitoring.

This high level, durable *w*Mel establishment was observed consistently throughout Niterói, with *w*Mel prevalence ≥ 95% in the *Ae. aegypti* population in every neighborhood in zones 1–4 that was monitored in 2023 (26 of 32 total neighborhoods), 44–53 months after the end of releases (Figure 3). Long-term monitoring collections also detected *Ae. albopictus* at a variable relative abundance compared to *Ae. aegypti* (Figure S1).

### 3.2. Sustained Suppression of Dengue Transmission in Niterói

**Figure 4:**
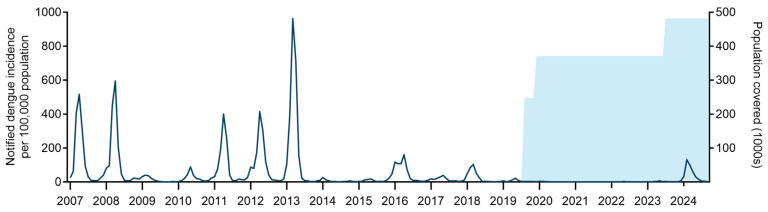
Dengue incidence in Niterói before, during and after phased *Wolbachia* mosquito releases. Dark blue line is the monthly incidence of dengue case notifications per 100,000 population in Niterói from January 2007 to September 2024. Blue shading indicates the cumulative residential population of the areas in which *Wolbachia* releases had been completed.

Dengue notifications data was available for ten years (2007–2016) prior to the first *Wolbachia* mosquito releases in Niterói, during which time a total of 43,488 dengue cases were reported in the city: an average of 4349 notified cases per year [annual range 366–11,619], corresponding to 913 cases per 100,000 people per year [annual range 75 to 2396/100,000 people]. By comparison, in the five-year period October 2019–September 2024 during which *Wolbachia* has been deployed throughout the entire urban area of Niterói, there were a total of 2193 dengue cases reported in the city: an average of 439 cases per year, corresponding to an average incidence of 91 cases per 100,000 people per year. Notably, 83% (*n* = 1820) of those cases were reported in 2024 during a period of unprecedented high dengue case incidence in the Americas, including Brazil, following four years of near-zero dengue incidence in Niterói (Figure 4).

There was a near absence of chikungunya and Zika case notifications in Niterói over the five years post-intervention: a total of 131 chikungunya and 34 Zika cases were notified between October 2019 and September 2024 (average annual incidence 5.4 and 1.4 per 100,000 population, respectively) (Figure S2).

Using ITS analysis to account for underlying temporal trends and the staggered implementation of the intervention across Niterói, notified dengue incidence was estimated to be 88.8% lower (95% confidence interval: 77.9 to 94.4%) following the completion of *Wolbachia* releases, compared to the 10-year pre-intervention period of 2007–2016. Dengue incidence was also significantly lower in the period during which releases were ongoing, compared to pre-release (90.2% reduction; 95%CI: 86.1 to 93.0%).

**Figure 5:**
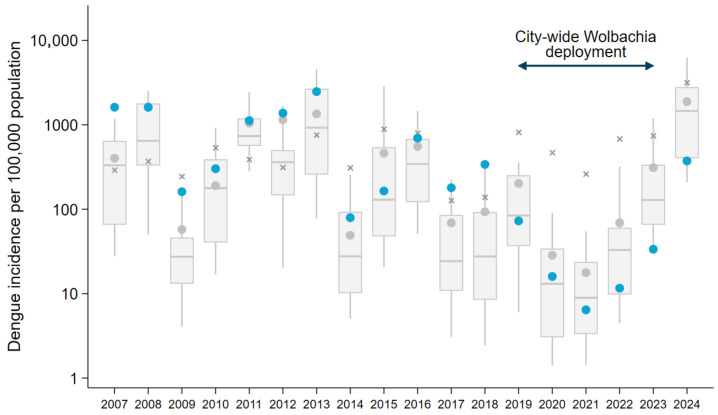
Annual dengue incidence in Niterói before, during and after *Wolbachia* deployment, compared to Rio de Janeiro state and nationally. The annual incidence of notified dengue cases per 100,000 population in Niterói (blue circles) is shown relative to the annual incidence among all cities (*n* = 28) in Rio de Janeiro state with population >100,000. Box plots show the median and interquartile range of the incidence in the 28 cities, and spikes show the 10th and 90th percentiles. The aggregate annual incidence in all of Rio de Janeiro state (gray circles) and all of Brazil (crosses) is shown for each year. The timing of phased city-wide roll-out of *Wolbachia* is indicated.

The inherent inter-annual and spatial variability in dengue epidemic cycles is a limitation in inferring intervention effects from a before-and-after analysis, or from comparisons with individual untreated municipalities. To inform an assessment of the dengue case incidence that could have been expected in Niterói in 2024 in the absence of the *Wolbachia* intervention—and thus the effectiveness of city-wide *Wolbachia* coverage in averting a larger outbreak in Niterói—we compared annual per capita dengue incidence in Niterói against the rest of Rio de Janeiro state and Brazil prior to, during and after *Wolbachia* deployments (2007–2024). Niterói has historically experienced high dengue incidence relative to other municipalities, ranking in the top 10 among the 28 large municipalities (≥100,000 population) in Rio de Janeiro state in all but one of the 12 years prior to the completion of area-wide releases in 2019. Dengue incidence in Niterói exceeded the Rio state average in ten of 12 years and exceeded the national average in seven of 12 pre-intervention years (Figure 5; Figure S3). This ranking has changed dramatically following *Wolbachia* deployments, with annual dengue incidence in Niterói 44–89% lower than the state average and 88–98% lower than the national average in every year since 2020 and Niterói ranking among the lowest incidence cities in the state each year since 2022 (Figure 5). Dengue incidence in Niterói in 2024 was 374 per 100,000 population (*n* = 1801 cases), compared to 1884 per 100,000 in all of Rio de Janeiro state and 3157 per 100,000 nationwide in Brazil.

Using the median and 75th percentile of the 2024 dengue incidence among all the large cities in Rio de Janeiro state to project a range of dengue cases that could reasonably have been expected in Niterói in 2024 in the absence of *Wolbachia*, we estimate conservatively that the *Wolbachia* intervention averted at least 5242 to 11,660 dengue cases in Niterói in 2024, corresponding to a reduction in case burden of between 74% and 87%.

## 4. Discussion

The *w*Mel strain of *Wolbachia* was durably established in local *Ae. aegypti* populations throughout the Brazilian city of Niterói by late 2023, making Niterói the first city in Brazil with citywide *Wolbachia* coverage. A four-year period of historic low dengue incidence in 2020–2023 followed *Wolbachia* deployment, before a relative increase in incidence was observed in Niterói in 2024 during an unprecedented dengue outbreak in Brazil and the region. However, case incidence in Niterói remained substantially lower than would be expected based on comparisons with historical dengue trends in Niterói and with all other large municipalities in Rio de Janeiro state and nationally. Our findings suggest that *Wolbachia* prevented at least three-quarters of the dengue case burden that may have otherwise occurred in Niterói in 2024, corresponding to thousands of cases averted. This extends existing evidence by showing that the public health benefits of successful area-wide establishment of *Wolbachia* are sustained even in a context of very high dengue transmission intensity.

A consistently high prevalence of *w*Mel (≥95%) was observed in the *Ae. aegypti* population in each of the 26 individual neighborhoods of Niterói monitored >4 years post-release, despite substantial initial variability of invasion levels within the first 12 months of post-release monitoring, and with no re-releases undertaken. This is in contrast to the neighboring city of Rio de Janeiro where, following similar initial variability in invasion levels, others have reported that *w*Mel prevalence in *Ae. aegypti* collected in 6 intervention neighborhoods declined dramatically in mid-2022 (~2.5 years post-release) and did not recover [28]. The authors hypothesize that a lower dessication resistance of *w*Mel-infected versus uninfected eggs led to a differential recovery of uninfected versus *w*Mel-infected *Ae. aegypti* populations following a crash in overall *Ae. aegypti* abundance in the dry, cool season in mid-2022, which coincided with a change in the larvicide used by public health teams. The same *w*Mel-*Ae. aegypti* line and production processes were used for the deployments in Niterói and Rio de Janeiro and the reasons for the different invasion trajectories in the two cities are not fully understood, but may reflect the increased complexity and insecurity of the urban environment in much of Rio de Janeiro, resulting in more spatial variability in the ‘effective dose’ of *w*Mel mosquitoes released and in their mixing with uninfected populations during the release phase.

In the 2023 expanded release area in Niterói, material for monitoring *w*Mel invasion was collected using ovitraps instead of adult mosquito collections, in order to integrate field monitoring with the routine vector surveillance activities undertaken by municipal health department staff. A switch to ovitrapping was also implemented in 2024 for long-term monitoring of the earlier release areas, but insufficient sample sizes were obtained from ovitraps in 2024 (<10 *Ae. aegypti* per zone per month) to enable inclusion in our analyses. While ovitrapping provides an opportunity to reduce costs by integrating *Wolbachia* monitoring with routine public health activities, additional work is required to validate the comparability of *w*Mel prevalence estimates from egg versus adult collections in the same location. Although a previous study in Brazil showed a high correlation between weekly *w*Mel *Wolbachia* frequency estimates based on screening of larvae obtained from eggs collected from ovitraps, and adult mosquito collections from BG-Sentinel traps [31]; these were based on relatively large weekly sample sizes (approximately 200 individuals for each method per week) and high density of traps (125/km^2^ and 188/km^2^, BG-Sentinel and ovitraps, respectively). Large scale monitoring of *Wolbachia* establishment using ovitraps needs to take into consideration the minimum overall and maximum number of samples per trap for screening, the spatial distribution of egg collections, and the storage conditions for eggs and larvae prior to *Wolbachia* PCR screening.

Monitoring of *w*Mel prevalence in *Ae. aegypti* populations throughout Niterói in 2025 will be important to understand whether the high level of *Wolbachia* establishment measured in late 2023 was sustained throughout the 2023/24 and 2024/25 summer periods. Density and prevalence of *w*Mel *Wolbachia* has been shown to decline at high temperatures under laboratory conditions [32], however these conditions may not accurately reflect the situation in the field. Field observations of the potential effects of elevated temperatures on *w*Mel *Wolbachia* establishment and durability have been limited. Semi-field studies in northern Australia where larvae were reared in semi-shaded locations where water temperatures reached up to 39 °C, showed that adult males had partially lost their ability to induce cytoplasmic incompatibility, and females had a greatly reduced egg hatch when crossed to infected males [33]. Field assessments during heatwave conditions in northern Australia found short term perturbations in *w*Mel infection prevalence (88%), but this recovered to be near 100% four months later [34]. In central Vietnam, the *w*Mel *Wolbachia* infection prevalence in mosquitoes was reduced during the hot season, as a result of reduced maternal transmission of *w*Mel from infected female mosquitoes [27]. In one site, this contributed to the loss of *w*Mel infection in the local *Ae. aegypti* populations while in another site it was transient with subsequent recovery to very high *w*Mel prevalence. The impact of elevated temperatures and other environmental effects on mosquito host fitness and the ability of *Wolbachia* to establish and persist in mosquito populations is therefore likely to depend on complex local environmental conditions. As a precaution, areas that experience extreme climatic conditions, including elevated summer temperatures or extended heatwave conditions, should be monitored to ensure the stability of *Wolbachia* in the local mosquito populations.

The occurrence of reported dengue cases in an area where *Wolbachia* has been deployed is not unexpected for several reasons. Firstly, the virus-blocking effect that *Wolbachia* (*w*Mel and *w*AlbB strain) confers on *Ae. aegypti* is not perfect and laboratory experiments have shown that some mosquitoes can develop infectious saliva (‘breakthrough infection’) despite the presence of *Wolbachia*, especially when the viral load in the blood meal is very high, and with a higher probability for DENV-1 than for other serotypes [35,36]. Secondly, even where area-level monitoring shows very high *Wolbachia* prevalence, there may remain pockets of wild-type *Ae. aegypti* sufficient to sustain limited local dengue transmission, especially when overall mosquito abundance is exceptionally high due to climatic or other factors. Importantly however, complete coverage and virus blocking by *Wolbachia* is not necessary to significantly reduce dengue incidence. Mathematical modeling predicts a significant and sustained reduction in dengue incidence in human populations even with incomplete virus blocking or *Wolbachia* coverage in the mosquito population [36,37,38], attributable to a *Wolbachia*-mediated reduction in the effective reproduction number during an outbreak (the average number of new infections arising from an infected individual). Consistent with this, our findings suggest that *Wolbachia* significantly reduced the peak of the epidemic curve and thereby the overall case burden in Niterói, but did not eliminate all transmission during a year of exceptionally high national transmission intensity. A reduction in case incidence to zero following *Wolbachia* deployments is furthermore not expected because notified dengue cases in Brazil and most other endemic settings are probable cases, reported on the basis of a clinical case definition [29] and often without laboratory testing, meaning an unknown proportion are febrile illnesses of another etiology, and because cases are notified by place of residence but may have acquired their infection in other locations. We note also the presence of *Ae. albopictus*, a competent vector of dengue viruses, throughout Niterói.

The challenge for assessing the real-world effectiveness of *Wolbachia* deployments from routine data sources across different settings lies in evaluating observed dengue case occurrence against what might have been expected in the absence of the intervention—i.e., the counterfactual—which is difficult to establish with certainty given the natural spatial and temporal variability in dengue epidemic cycles. These constraints are not specific to *Wolbachia*-based dengue control, they are common to many public health evaluations where a well-matched untreated control group is unavailable. In such scenarios, interrupted time series analysis—using multiple consecutive pre- and post-intervention observations in a single population and explicitly incorporating time—has been shown to be a powerful quasi-experimental study design for estimating intervention effect [30,39]. The internal consistency in our estimates of *Wolbachia* intervention effect from uncontrolled ITS (within-population analysis) and from a comparison of annual dengue incidence in Niterói versus other Brazilian cities (between-population analysis)—as well as the external consistency with intervention effects reported in other settings and the biological plausibility given very high *w*Mel prevalence—provides reassurance that these findings are robust to potential confounding effects. However, as for any real-world observational study, we cannot fully exclude the possibility that secular trends, artifacts of healthcare-seeking or reporting behaviors, or other factors contributed to the observed trends in dengue incidence in Niterói over time.

This reduction in notified case incidence attributable to *Wolbachia* deployments in Niterói translates into a considerable broader impact at individual, health system and societal levels. The annual direct and indirect costs of dengue in Brazil were estimated at between US$517 and 1688 million in the years 2009–2013 [40], and the average cost per dengue case has been estimated at US$532 in 2013 dollars [41]. Hospitalization status is not well-recorded in the publicly available SINAN dengue notifications data, but previous studies have reported hospitalization rates of dengue cases in Brazil ranging from 3 to 4% up to 13–30% [40,41,42]. Even at the lower end of this range, this indicates a substantial alleviation of dengue-related hospital admissions in Niterói in 2024 as well as the associated healthcare costs of both hospital and outpatient care, the indirect household and societal costs of ill-health and care-giving, and the financial costs associated with insecticide applications by municipal health staff in response to reported cases.

In response to the rapid expansion in the population at risk of dengue in Brazil and in the frequency and magnitude of dengue epidemics, the Brazilian Ministry of Health published in January 2025 a revised national contingency plan for dengue, chikungunya and Zika which identifies the phased expansion of *Wolbachia* releases and other new vector control technologies as a priority activity, alongside the roll-out of dengue vaccination programs and the strengthening of capacity in surveillance, outbreak response and clinical case management [43]. Our results—and the scale of the dengue problem in Brazil and the region—highlight the importance of an integrated approach that employs all available solutions, with *Wolbachia* implemented as a complementary tool alongside other conventional and innovative vector control methods, and with integrated planning and oversight to maximize the individual and combined effects of different interventions [44,45]. Monitoring of entomological and epidemiological outcomes of *Wolbachia* deployments in Brazil, including in cities where *Wolbachia* establishment has been less homogenous, is critical for a balanced evaluation of *Wolbachia* as a public health intervention and for identifying operational or ecological factors associated with suboptimal establishment and/or durability [28]. An ongoing randomized controlled trial of *Wolbachia* deployment in Belo Horizonte will add to the evidence-base [46], as will evaluations of local dengue vaccination campaigns and the comparative effectiveness and cost-effectiveness of *Wolbachia* and vaccination together or alone. Finally, planning for and investment in the necessary infrastructure, technical capabilities, financing and operational models for scaled *Wolbachia* implementation and monitoring is essential for effectively integrating these new evidence-based interventions into disease control programs in Brazil and the many other countries facing a rising burden of dengue.

## Data Availability

All data underlying these analyses is available at the following links: Niteroi monthly *w*Mel monitoring data by neighborhood 2017–2024 (https://doi.org/10.26180/28737803.v1). Niteroi notified dengue, chikungunya and Zika monthly case counts 2007-September 2024 (https://doi.org/10.26180/28737812.v1). Annual notified dengue incidence in Brazilian municipalities (SINAN database), 2007–2024 (https://doi.org/10.26180/28737815.v1).

## Supplementary Materials

The following supporting information can be downloaded at https://www.mdpi.com/article/10.3390/tropicalmed10090237/s1. Table S1: Summary of *w*Mel-*Ae. aegypti* releases and monitoring in Niterói. Figure S1: Relative abundance of *Ae. aegypti* and *Ae. albopictus* in long-term entomological monitoring in Niterói during and after *w*Mel-infected *Ae. aegypti* releases. Counts of *Ae. aegypti* (blue lines with circle markers) and *Ae. albopictus* (orange lines with triangle markers) caught by BG traps (solid lines and filled markers) or by mechanical aspirators (dashed lines and open markers) were aggregated for each release zone each month. Release periods are indicated with gray shading. Zone 5 is excluded because all monitoring was performed using ovitraps, not adult collections. One outlier observation is omitted from the zone 1 graph (23 *Ae. albopictus* in one aspirator collection in February 2022) and one from the zone 2 graph (583 *Ae. albopictus* in 30 aspirator collections [mean 19.53 per collection] in August 2023) to improve visibility of the remaining data. Figure S2: Monthly incidence of notified chikungunya (solid line) and Zika (dashed line) cases per 100,000 population in Niterói, before, during and after phased Wolbachia mosquito releases (January 2015 to September 2024). Blue shading indicates the cumulative residential population of the areas in which *Wolbachia* releases had been completed. Figure S3: Annual dengue incidence in Niterói before, during and after *Wolbachia* deployment, relative to other dengue-affected cities in Brazil. The annual incidence of notified dengue cases per 100,000 population in Niterói (blue circles) is shown relative to the annual incidence among all cities (n = 316) in Brazil with population >100,000 and a non-zero number of dengue cases notified each year since 2007. Box plots show the median and interquartile range of the incidence in the 316 cities, spikes show the 10th and 90th percentiles of the incidence in the 316 cities, and gray circles show the aggregate incidence for all of Brazil. The timing of phased city-wide roll-out of *Wolbachia* is indicated.

## Abbreviations

The following abbreviations are used in this manuscript:

SINAN	Sistema de Informação de Agravos de Notificação
PCR	Polymerase chain reaction
DENV	Dengue virus
ITS	Interrupted time series
