# Supplementary material for: Long-Term Durability and Public Health Impact of City-Wide wMel Wolbachia Mosquito Releases in Niterói, Brazil, During a Dengue Epidemic Surge
Source: Trop Med Infect Dis. 2025 Aug 25;10(9):237. doi: 10.3390/tropicalmed10090237 (PMC12474452; doi:10.3390/tropicalmed10090237)
Supplement: Supplementary file 1 [file tropicalmed-10-00237-s001.zip › tropicalmed-3779628-supplementary.pdf]

Supplementary Table S1. Summary of *w*Mel-*Ae. aegypti* releases and monitoring in Niterói.

Zone	Population (2022)	Total Area (km²)	Release Area (km²)	# Release Periods	First Release	Last Release	Estimated mosquitoes released (millions)	# of Traps* (& after reduction)	Trap density* per km2 (& after reduction)	Start Monitoring	End Monitoring
**1**^	23,592	8.5	3.5	2	Feb 2017	Dec 2019	2.64	138 (49)	39 (14)	Feb 2017	Oct 2023
**2**	68,246	51.3	18.9	2	Jun 2017	Jul 2019	12.84	302 (229)	16 (12)	Jul 2017	Oct 2023
**3**	177,722	12.2	9.4	3	Nov 2017	Jul 2019	12.61	169	18	Dec 2017	Aug 2023
**4**	101,119	10.9	8.1	1	Sep 2019	Dec 2019	6.17	140	17	Oct 2019	Sep 2023
**5**	111,070	50.5	16.2	1	Nov 2022	Jul 2023	21.29	105	6	Jan 2023	Sep 2024
**Total**	481,749	133.5	56.1				55.55				

*In zones 1-4 *Wolbachia* monitoring used adult mosquitoes collected with BG traps (2017-2020) or aspirators (2021-2023), and in zone 5 used larvae reared from ovitrap collections.

^Release zone 1 includes the Jurujuba neighbourhood where pilot releases were conducted in 2015–16 (1), for all metrics except 'Estimated mosquitoes released' which includes only the expanded releases in zone 1 beginning in February 2017; the month that BG traps were first installed in zone 1 also excludes the pilot release period.

Note: release area comprises all urban or constructed areas in the zone, but excludes green non-constructed areas, which are less favourable habitats for *Ae*. *aegypti*. The number and density of BG traps was reduced in parts of zone 1 and zone 2 in order to reduce monitoring costs, once releases were completed and neighbourhood-level *w*Mel prevalence was >60% in 3 consecutive monitoring events measured at least 4 weeks after the conclusion of releases.

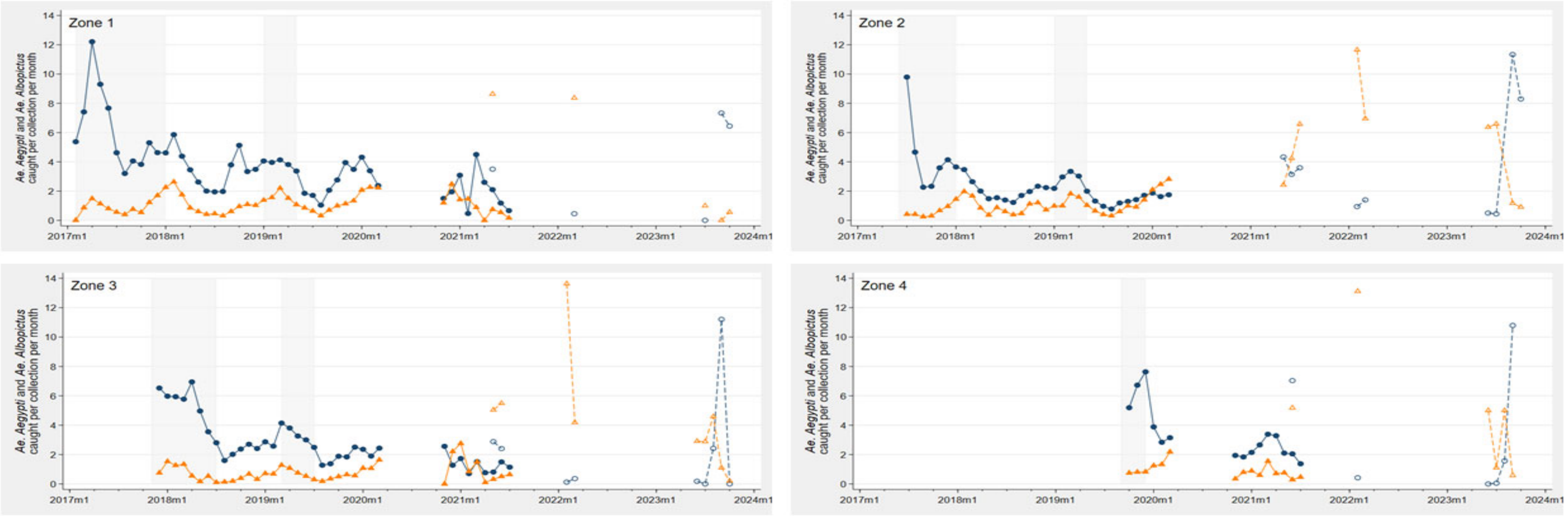

Supplementary Figure S1: Relative abundance of *Ae. aegypti* and *Ae. albopictus* in long-term entomological monitoring in Niterói during and after *w*Mel-infected *Ae. aegypti* releases. Counts of *Ae. aegypti* (blue lines with circle markers) and *Ae. albopictus* (orange lines with triangle markers) caught by BG traps (solid lines and filled markers) or by mechanical aspirators (dashed lines and open markers) were aggregated for each release zone each month. Release periods are indicated with grey shading. Zone 5 is excluded because all monitoring was done using ovitraps, not adult collections. One outlier observation is omitted from the zone 1 graph (23 *Ae. albopictus* in one aspirator collection in February 2022) and one from the zone 2 graph (583 *Ae. albopictus* in 30 aspirator collections [mean 19.53 per collection] in August 2023) to improve visibility of the remaining data.

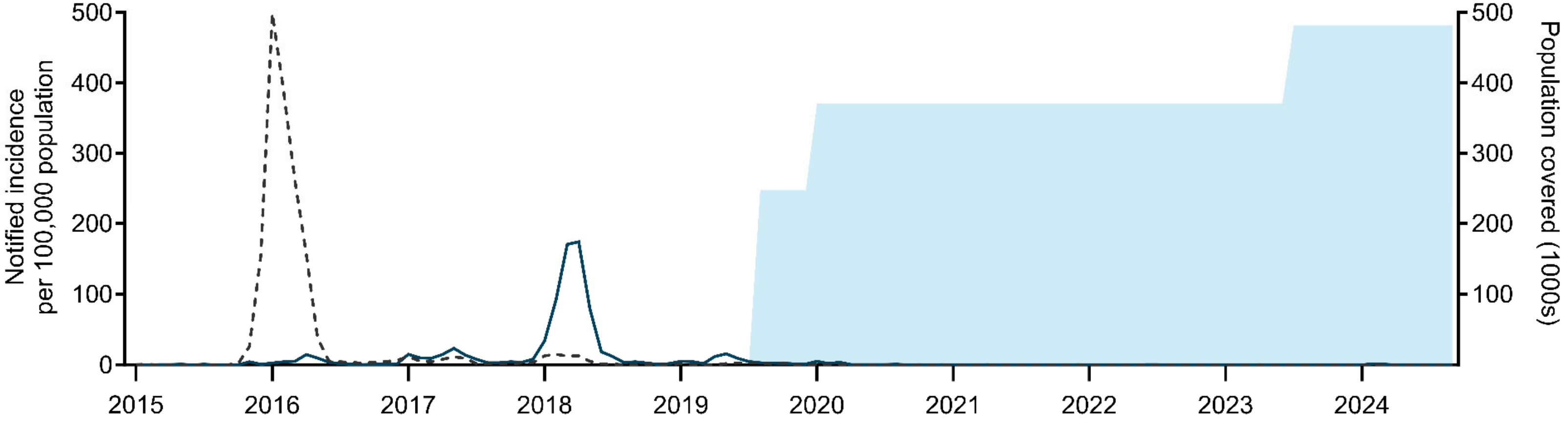

Supplementary Figure S2: Monthly incidence of notified chikungunya (solid line) and Zika (dashed line) cases per 100,000 population in Niterói, before, during and after phased *Wolbachia* mosquito releases (January 2015 to September 2024). Blue shading indicates the cumulative residential population of the areas in which *Wolbachia* releases had been completed.

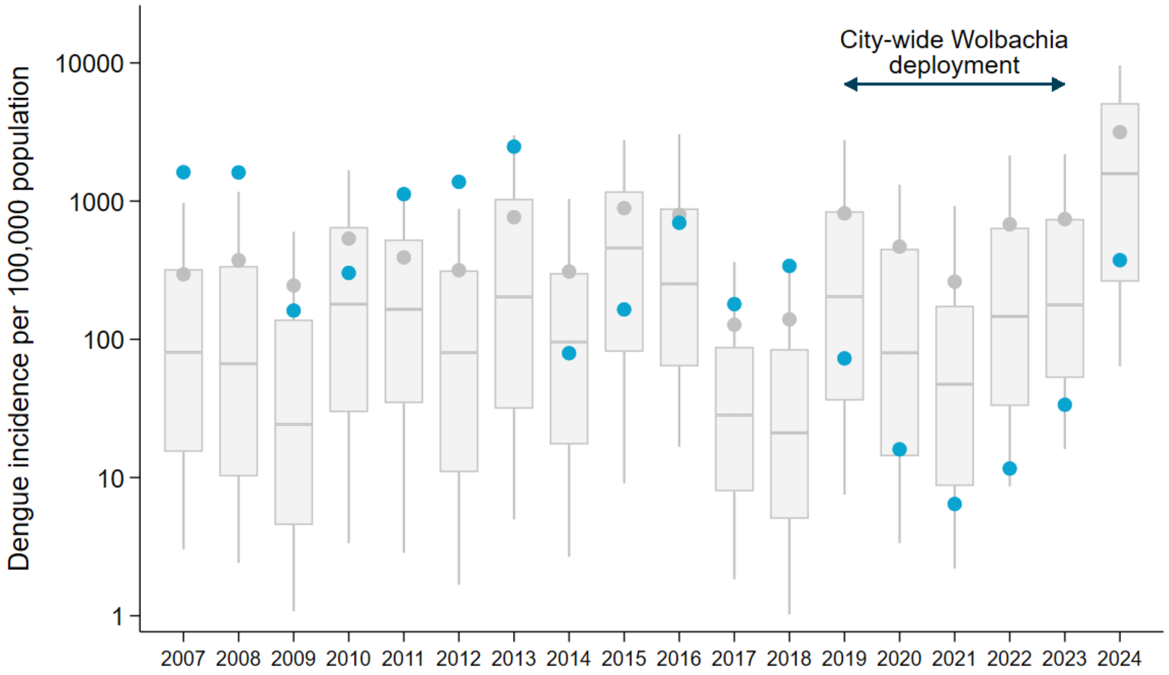

Supplementary Figure S3: Annual dengue incidence in Niterói before, during and after *Wolbachia* deployment, relative to other dengue-affected cities in Brazil. The annual incidence of notified dengue cases per 100,000 population in Niterói (blue circles) is shown relative to the annual incidence among all cities (N=316) in Brazil with population >100,000 and a non-zero number of dengue cases notified each year since 2007. Box plots show the median and interquartile range of the incidence in the 316 cities, spikes show the 10th and 90th percentiles of the incidence in the 316 cities, and grey circles show the aggregate incidence for all of Brazil. The timeline of phased city-wide roll-out of *Wolbachia* is indicated, including the release period (dotted line) and post-release period (solid line) in zones 1-4, covering 70% of the urban population, and the expansion of releases to the remaining urban areas (thicker line).

Reference

1. Gesto JSM, Ribeiro GS, Rocha MN, Dias FB, Peixoto J, Carvalho FD, et al. Reduced competence to arboviruses following the sustainable invasion of *Wolbachia* into native *Aedes aegypti* from Southeastern Brazil. Sci Rep. 2021 May 11;11:10039.
